# Assessment of the Functional Form of the Relationship between Balance Control and Physical Activity Regarding Demographic, Anthropometrical, and Eye Impairment Explanatory Covariates in 9- to 11-Year-Old Children: Results of Polynomial and Cluster Analyses

**DOI:** 10.3390/biology11111663

**Published:** 2022-11-14

**Authors:** Jarosław Domaradzki, Monika Modrzejewska, Dawid Koźlenia, Teresa Zwierko

**Affiliations:** 1 Unit of Biostructure, Faculty of Physical Education and Sport, Wroclaw University of Health and Sport Sciences, 51-612 Wroclaw, Poland; 2 II Department of Ophthalmology, Pomeranian Medical University, 70-111 Szczecin, Poland; 3 Laboratory of Kinesiology, Functional and Structural Human Research Center, Institute of Physical Culture Sciences, University of Szczecin, 70-240 Szczecin, Poland

**Keywords:** functional development, children, balance control, myopia, biological age, functional form

## Abstract

**Simple Summary:**

The shape of an association between balance control and physical activity (PA) concerning sex, body mass index (BMI), calendar age, biological age, and myopia is not a well-described issue. Therefore, this study aimed to establish the relationship between balance control and PA in the presence of the above-mentioned demographic, anthropometric, and eyesight impairment factors. In our study, 47 boys and 58 girls aged 9–11 years participated. The results showed that only BMI and myopia affected the relationship between balance and PA, demonstrating three kinds of shapes of the relationships. Non-myopic girls with a low BMI and less maturity constantly improved their balance control with increased PA volume. Early-matured boys with a BMI slightly over average or in a normal range achieved a plateau in PA level for balance-control development. Myopic boys and girls with higher BMIs than their peers but maturing at an average pace had a peak value of PA, which is the threshold for the volume of PA when the stimulating effect starts. Future studies should be focused on evaluating the causal relationship between balance control and PA using other explanatory variables.

**Abstract:**

Explaining the causal and functional relationship between balance control and physical activity (PA) when comparing demographic, anthropometric, and eyesight impairment is uncharted. This study aimed to assess the shape of the relationships between balance control and PA and to verify the usefulness of explanatory variables (sex, chronological age, biological age, myopia, and BMI) in explaining the formation of functional forms between both abovementioned variables. The current contribution evaluated data from 9–11-year-old children (47 boys, 58 girls) and attempts to explain the shape of the relationship between the overall stability index and PA synthetic index, sorting children into clusters depending on their balance features and PA and comparing the separated groups in terms of explanatory variables. The analysis demonstrates four larger clusters that displayed distinct functional relationships. Only BMI and myopia turned out to be useful in explaining cluster memberships. Children in clusters with a linear-constant decline functional form were mostly non-myopic girls, thin, and less mature. Meanwhile, it becomes clear that children with an L-shape were myopic, early-maturated boys with a BMI in the middle of the range for normal weight. The pattern of an inverted U-shaped functional form was distinctive for myopes with rather high BMIs compared to their peers but normally matured.

## 1. Introduction

There is a growing interest in the study of physical activity (PA) and fundamental motor skills (FMS), as well as biological factors affecting its relationship in children and adolescents. FMS are the building blocks that lead to the acquisition of more complex movement sequences required for adequate participation in physical activities in children, adolescents, and adults [1]. Balance is an important element of FMS acquired during a child’s development and is a functional prerequisite for more complex motor skills [2,3]. The interaction of multiple sensorimotor processes based on vestibular, visual, and proprioceptive information determines balance control [4]. However, the particular contributions of these inputs vary during ontogenesis [5].

It has been generally accepted that PA has protective and stimulatory effects on a child’s functional status, i.e., better physical and cognitive health [6]. In particular, PA is associated with many positive health outcomes during the early years and contributes to a child’s physical, cognitive, and social development [7]. Furthermore, studies have shown that PA and FMS, like a balance, have reciprocal interactions and a number of health-related factors and motor competencies that influence PA participation during childhood [8]. Several studies have reported a cause–effect relationship between FMS and PA [9,10], indicating that PA could stimulate functional development during childhood, consequently resulting in PA involvement at a later age. This cause–effect relationship between FMS and PA seems to be very important in the context of childhood health conditions, such as poor vision in myopia.

Myopia is a major public health ocular disorder that commonly develops in school-age children [11]. Nowadays, myopia affects nearly 30% of the world’s population, and this number is anticipated to rise to 50% by 2050 [12]. Myopia risk factors include age, race, obesity, ethnicity, urbanization, educational level, and occupation [13,14]. Among many environmental factors, the incidence of myopia and its progression in children is associated with the trend of spending considerable amounts of time using computers and smartphones [12,15], as well as reduced PA and time spent outdoors [16,17]. It has been suggested that the protective effects of time spent outdoors result in a reduction in spasms of accommodation, increased daily light exposure, and release of dopamine from the retina inhibiting the excessive lengthening of the eye (axial elongation), which is the structural basis of myopia [18]. Therefore, increased time outdoors is beneficial in preventing the onset of myopia, as well as slowing the myopic shift in refractive error, but it does not stop the progression of myopia [19]. PA has protective and stimulatory effects on many aspects of a child’s functional status and health [20]. Regarding the relationship between time spent outdoors and myopia, it was reported that an increase of 60 min spent outdoors each day can reduce the risk of myopia development by approximately 13.3% [21]. Unfortunately, studies have identified a lower PA participation in children with myopia compared to non-myopic children [22,23]. Moreover, one of the contributing factors of physical inactivity in myopic children may be their parents’ perception that their child has a poorer level of sporting abilities [24,25], which contributes to a decreased participation in organized forms of PA, i.e., participation in sports clubs and extracurricular PA [23].

A recent study showed that functional status assessed using a dynamic balance task was worse in myopic children compared to non-myopic children, and PA moderated the relationship between myopia and a child’s functional status [26]. Research in this area is becoming more important, as myopia is becoming more prevalent and can cause serious pathological complications in later life (i.e., open-angle glaucoma, retinal detachment, myopic macular degeneration, and cataracts) [27].

There is evidence from biological studies that prove a curvilinear (second or higher term) rather than a rectilinear relationship between biological features [28]. In studies concerning motor development, the same conclusions were drawn years ago [29,30] and have been validated recently [31,32]. Thus, studying the relationship between PA and balance control is strongly justified. Another problem is identifying the patterns in growth or relationships between factors stimulating or limiting motor development. Identification of the variables related to potential patterns is another way of scientifically evaluating these relationships. Statistical methods used to classify individuals (cluster analyzes) are very useful and widely used in such analyses [33,34]. Classifying and explaining the causal and functional relationships between factors that affect PA engagement in children is important in aiding the design of more effective interventions. Understanding the possible shape of the interaction between PA and FMS is essential when planning PA. The dependence can be linear (constant increase or decrease in motor functions with increased PA), polynomial (U-shaped or inverted U-shaped that suggests a peak as well as optimal and suboptimal values), or hyperbolic (with a plateau during a specific range of the PA). The study of the possible shape of relationships is necessary and important, yet the relationship mentioned above in children is related to progressive growth phenomena. To date, determinants of PA in children have been investigated to some extent [35,36]. Meanwhile there are very well-known and studied factors that affect PA, such as sex—boys are usually more likely to take part in PA than girls [37,38,39] and chronological age (CA)—schoolchildren are more likely to take part in PA than older ones [37,39,40]. However, there is still a lack of studies taking into account biological age in PA engagement. Some studies have shown that younger children are more likely to participate in PA than pubescent and adolescents, but the results are inconclusive [41,42]. In our opinion, there is a lack of research on the relationship between PA, myopia, biological age, and FMS, resulting in a knowledge gap that needs further exploration.

The moderating role of PA on the relationship between myopia and functional status in primary school children was published in another paper [26]. This paper deeply studies the real shape of the association between PA and balance control in prepubescent children. Therefore, the main aim of this contribution is to shed more light on the shape of the relationship between PA and balance control. In addition, more attention should be given to comparisons regarding specific variables (demographic, anthropometric, and myopia) that might influence the functional forms of PA and balance control. Specifically, the present study answers the following detailed questions: (1) What is the functional form of the relationship between PA and balance control? Additionally, does adding higher-order terms for PA improves a model’s fit? (2) Can children be classified into clusters that follow similar patterns? (3) Which variables determine the classification of children into clusters? How are clusters characterized with respect to demographic, anthropometric, and myopic variables?

Our initial hypotheses stated that the association between balance control and PA will have no first-order character, but higher-order regressions will explain a better relationship. There is a significant difference in balance control between participants, and they can be classified into clusters that follow similar patterns. Clusters can be characterized with respect to some demographic and anthropometric variables.

## 2. Materials and Methods

A detailed description of the studied groups of children, methods of measurements, and procedures are presented in Modrzejewska et al. [26].

### 2.1. Power Calculations

A power calculation was conducted before recruitment. It was calculated that 105 participants in total would be required to detect between-group differences in the measured outcomes. Although it was calculated for a two-way ANOVA (presented in our earlier work), it is also valid for comparing the four clusters of participants identified in this work. Based on an 85% total power, an α level of 0.05, and a minimum effect size of 0.35, it was calculated that 105 participants would be required to detect between-group differences in the outcome values. To calculate the sample size, the G*Power tool (Heinrich-Heine University, Düsseldorf, Germany) was used [43].

### 2.2. Participants

Eye-screening tests were conducted on 1518 schoolchildren aged 9–11 living in Szczecin, Poland between the years of 2017 to 2019. In the group of 1518 children, post-cycloplegia myopia was confirmed in 255 children (16.8% of all subjects). Fifty-two (27 girls and 25 boys) children from the group of 255 with myopia and 53 non-myopes (33 girls and 20 boys) volunteered for the functional status assessment. All participants completed a balance test and PA questionnaire within one month of the eye examination. The screening portion of our study included all children enrolled in a primary school aged 9–11 with no other exclusion criteria. In the next stage of this study, children meeting the following inclusion criteria were identified: the spherical equivalent of refraction ≤−0.5 SD for myopes and >−0.5 SD for non-myopes, ocular alignment measured by the Maddox test within the normal range, no deviations in the form of overt and latent strabismus, presence of foveal fixation, and in good health and physical condition. The exclusion criteria included illness or injury that prevented completion of the balance test. Refraction was obtained using a hand-held Retinomax 3 autorefractometer (Righton, Tokyo, Japan) with and without cycloplegia using 1% tropicamide administered every 10 min for a total of three times. Table 1 summarizes the descriptive demographic, anthropometric, and clinical characteristics of the children. Clinical variables presented include spherical equivalent values, i.e., the tested refraction in children immediately before the administration of agents paralyzing accommodation and after the administration of drops paralyzing accommodation. This was necessary to determine whether the value of myopia is a refractive error—a permanent one, i.e., one that is negative before and after the administration of cycloplegia indicating myopia. Therefore, we were able to prove that in our study group, all children who qualified for the current study were myopic.

#### 2.2.1. Biological Age—Age at Peak Height Velocity (APHV)

Variations in the rate of growth, which indicates the moment of maturity in both sexes for myopic and healthy children, were assessed using the formulas proposed by Moore et al. [44]. The age at peak height velocity (APHV) was predicted using sex-specific regression equations: girls—maturity offset (MO) = −7.709133 + (0.0042232 × (age × height)); boys—MO = −7.999994 + (0.0036124 × (age × height)). APHV was calculated as CA—MO for each individual. The predicted MO is defined as the time before/after the APHV, and the predicted APHV was the CA minus the predicted offset [45]. Thus, MO is used to determine how far away an adolescent is from their peak height velocity (PHV) growth spurt.

#### 2.2.2. Balance Task

The Biodex Balance System SD (Biodex Medical Systems Inc., Shirley, NY, USA) was used to evaluate balance control. Balance was measured during a dynamic task (i.e., single leg stance on the preferred leg). The total duration of the balance task was 80 s (3 trials of 20 s with a rest interval of 10 s between each trial). For all trials, the children were tested barefoot. During the test, children looked straight ahead with their arms folded across their chests. The overall stability index (OSI, measured in degrees) was analyzed. The OSI represents fluctuations around zero, which was established prior to testing and when the platform was stable [46]. The intraclass correlation coefficients applied to OSI were 0.85 during static conditions and 0.77 during dynamic conditions, respectively [47].

#### 2.2.3. Physical Activity

PA was measured using the Polish version of the questionnaire items according to the WHO European Childhood Obesity Surveillance Initiative (COSI) [48]. Questionnaires were completed by the parents or guardians (a proxy-respondent tool). Information regarding the child’s PA consisted of distance from school to home, transport from/to school (e.g., by bus, car, foot, etc.), participation in sports clubs and extracurricular PA per week (in hours), time spent participating in PA during the child’s leisure time, time spent on homework, and sedentary behaviors (screen time). The database was prepared based on the WHO COSI Guidelines on Data Processing and Cleaning. There are several close-ended questions in the COSI questionnaire. Respondent answers were ranked and linked to result in points (starting from 1 and increasing together with categories of the answer). Some of the questions were classified as stimulants, whereas others were deterrents for PA. In this study, a synthetic index of PA (PASI) was calculated. It was constructed based on the results of each answer in the questionnaire. First, raw points were scaled (the transformed variable had a mean of 0 and a standard deviation of 1). The nature of the variables (stimulants and deterrents) was taken into account. Next, all questions representing the different components of PA were accumulated into a common, agglomerated index, referred to as the agglomerated PASI. The agglomerated PASI was created using a Multidimensional Comparative Analysis (MCA) [49]. A detailed procedure for calculating PASI has been presented elsewhere [26].

### 2.3. Strategy of Analysis

This section outlines which steps in the analysis process were taken to solve the problems stated in the aims of this work.

(1) The balance control variable (OSI—dependent variables, DVs) was regressed on the physical activity synthetic index (PASI—independent variables, IVs). Three separate regression functions were calculated: PASI, PASI^2^, and PASI^3^. The models were used to define the functional relationships between the volume of PA and balance control.
(2) The peak or minimum–maximum values were indicated for best-fitted models (if the best-fitted model was a polynomial function).
(3) Classification of the participants based on OSI and PA results was conducted with the hierarchical Ward-clustering method and Euclidean distances.
(4) To explain cluster membership, demographic, anthropometric, and eyesight impairment variables were employed.

### 2.4. Statistical Analysis

The Shapiro–Wilk test was used to evaluate the normality of data distribution, and the results for all continuous variables failed to reject the null hypothesis; thus, all variables were assumed to follow a normal distribution. Descriptive statistics of anthropometric measures and functional features have been presented elsewhere [26]. All continuous data were presented as means, standard deviations, and 95% confidence intervals (CI). Qualitative variables were presented as numbers and percentages.

The balance control variable, the OSI, was regressed on PASI. Three forms were calculated: simple linear regression (PASI), polynomial squared regression (PASI^2^), and polynomial cubic regression (PASI^3^) for the whole group of participants. Statistics evaluating model fit (R^2^) and regression coefficients were calculated. By doing so, we attempted to find the most accurate functions to explain the associations. The measurements were calculated using R-squared.

To find out the maximum or minimum value of quadratic polynomial regression (peak value—PASI for best or worst functional results), this formula was used:

$$x=-\frac{b}{2a}$$

where *b* is the coefficient for the linear part, and *a* is the coefficient of the quadratic part.

To find out the extremums (if they exist) in the cubic function, there was a need to indicate points (*x*_1_, *y*_1_) and (*x*_2_, *y*_2_). These formulas were then used:

$$x_{1}=\frac{-2b_{2}+\sqrt{\Delta }}{6b_{3}},$$

$$y_{1}=f/x_{1}/,$$

$$x_{2}=\frac{-2b_{2}-\sqrt{\Delta }}{6b_{3}},$$

$$y_{2}=f/x_{2}/$$

where Δ ≥ 0, $\Delta =4b_{2}{}^{2}-12b_{3}b_{1}$.

Based on the curve drawn for the cubic regression model, the peak value was calculated only for the extremums that represented age-related “template” values for the best results on the functional test.

To classify children based on OSI and PASI results, a hierarchical Ward clustering was applied. There are different linkage methods for combing clusters in agglomerative procedures (e.g., single linkage, complete linkage, centroid method, etc.). Unlike the others, instead of measuring the distance directly, Ward’s method analyzes the variance between clusters—it is based on the minimum variance criterion (it minimizes the total within-cluster variance) [50]. This method is said to be the most suitable method for quantitative variables [51]. Distance measurements were performed using the Euclidean distance formula. Before aggregation, the results of the OSI were normalized on mean = 0 and standard deviation = 1.

Mojena’s method was used to decide how many clusters should be formed in the analysis (what is the critical distance value by which class joining should be stopped).

Variables used for cluster membership were qualitative (sex, myopia presence) and quantitative (CA, biological age—APHV, and body mass index (BMI)). To assess the relationship between qualitative variables and the separation of the participants into clusters, χ^2^ tests were conducted. In the case of quantitative variables, ANOVA followed by Tukey’s post hoc test were used. This part of the analysis aimed to explain why there were differences in the functional form between OSI and PASI for clusters obtained during cluster analysis. Statistical significance was set at an α-value equal to 0.05. Statistica version 13.0 (StatSoft Polska, Cracow, Poland, 2022) was used for data analysis.

## 3. Results

Detailed descriptive statistics and basic comparisons between the groups of children were published elsewhere [26]. There were significant differences in balance control between myopic and non-myopic children with low and moderate levels of PA. At the same time, there were no significant differences between children with high levels of PA. High PA levels had a positive impact on balance control in myopes. Results showed that PA altered the relationship between myopia and functional status.

The first step in this analysis was to assess the relationship between the OSI and PASI for all children. Table 2 presents the regression coefficients and statistics of the model’s fit (R^2^, *p*-values). This step gives a general overview and insight into the global relationship patterns between PA and OSI. It allowed for an evaluation of the importance of PA on balance-control development in the prepuberty phase of growth in children. Three forms of regression were conducted to receive detailed impressions. In the first model, only PA was analyzed. In the second and third, PA^2^ and PA^3^ were used, respectively. This procedure allowed us to check whether adding a higher-order term for PA improved the model fit. Results are displayed in Table 2. The results clearly suggested that adding a higher term (PA^2^) for PA did indeed improve the model fit. The function F2 with the two terms received the highest and most significant R^2^ statistics (R^2^ = 0.092), which identified a curvilinear relationship between OSI and PASI.

**Figure 1:**
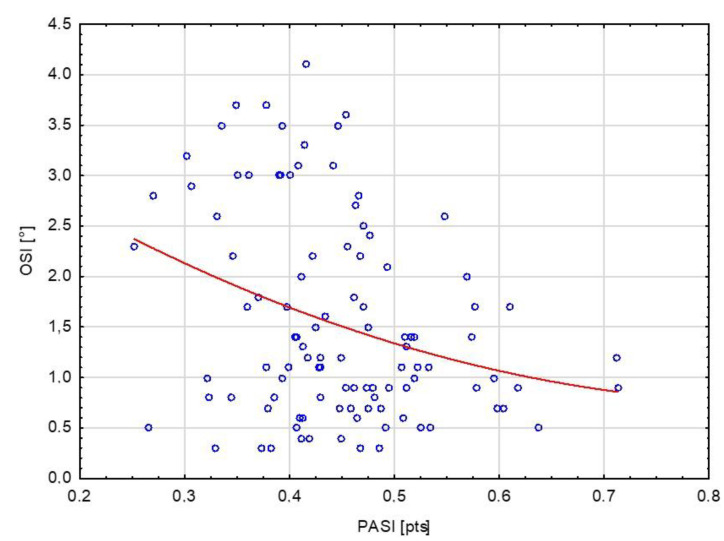
The functional form between physical activity and balance control for all participants.

The global functional form is visualized in Figure 1. The left side of the regression curve was steep. A linear decline was observed, which reached a minimum of 0.6–0.7 normalized PA points. It confirmed decreases in the OIS results (improvement in balance control) with an increased PA until a specific level of PA, after which further performance improvements were no longer as intense with a tendency toward stability. This showed that raising PA to a volume above the second tercile, above the average volume of PA (for results obtained from the study group), permanently raises the balance-control level.

In contrast, dependence is reduced after reaching a certain optimal level of PA. Using the formula presented in Section 2, the peak value to achieve the best balance control was precisely predicted. A predicted PA value of 0.88 points as the stabilization threshold confirms that the limit for improvement in the OSI concerning PA is distant.

**Figure 2:**
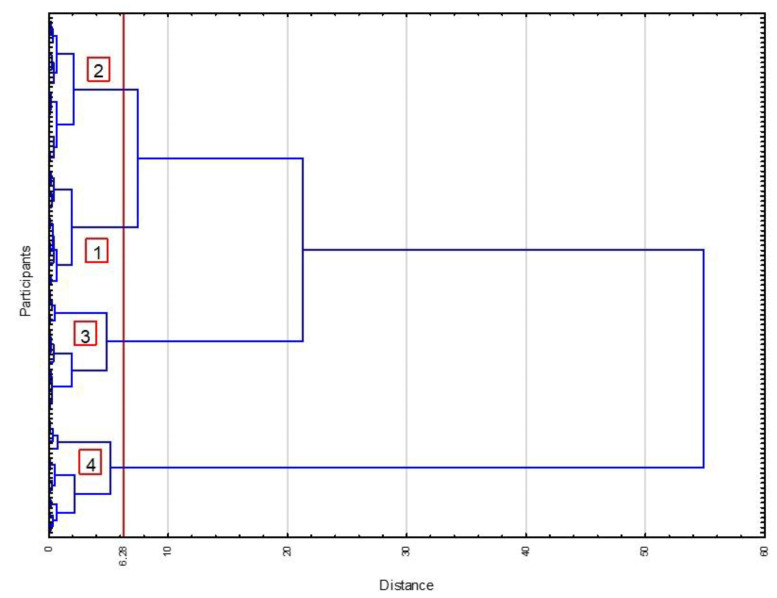
Dendrogram created by the clustering procedure. 1–4—clusters of the participants agglomerated based on computed regression coefficients for overall stability index regressed on physical activity (PASI, PASI^2^, PASI^3^); distance—Euclidean distance computed as a measurement of the similarity of the participants inside each cluster and dissimilarity of the participants between clusters.

The next step of the analysis was the agglomeration of participants based on OSI and PASI results. Euclidean distance was chosen as a measurement for (dis)similarity. A hierarchical Ward-clustering method with Euclidean distance was computed for similarity measurements. The result was a dendrogram with four clusters identified at a distance of 6.28 (Figure 2).

**Figure 3:**
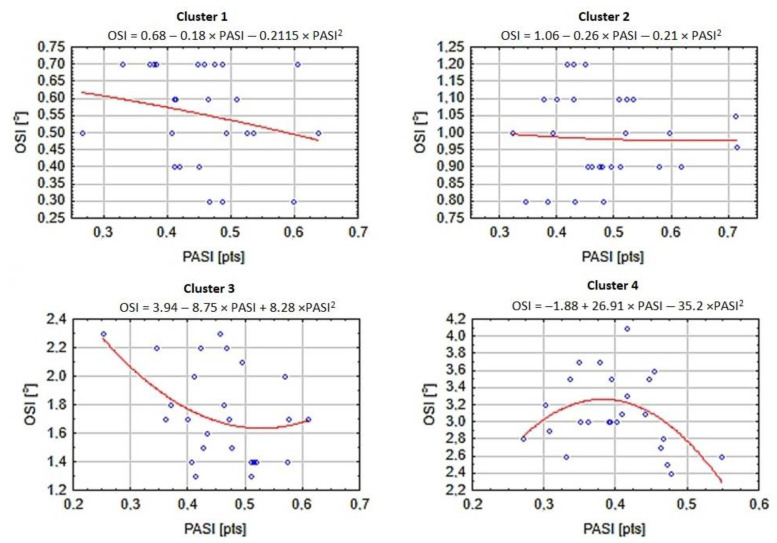
The functional form between PA and OSI by cluster.

The first two clusters were very similar and joined into a larger cluster close to the separation line. There were 25 participants in the first cluster and 30 participants in the second cluster. The third cluster (*n* = 26) was joined at a distance that was over twice as long, suggesting huge differences between the third cluster and the first and second clusters. However, the most dissimilar was the fourth cluster (*n* = 24), linked to all previous clusters at an approximate distance of 55. The internal homogeneity inside each cluster was high, but the similarities between clusters were very low. Therefore, there is a potential chance to receive different results for the functional form in each cluster. Thus, the functional form between PA and OSI for each cluster was calculated. Results are presented in Figure 3. Fractional-polynomial prediction plots were utilized.

As predicted, all functional forms were different between clusters. However, the most similar curves were cluster 1 and cluster 2. The shape was more close to linear than clearly curvilinear. Both lines showed a decline and constant changes in the OSI together with PASI. The plot for cluster 2 was less steep than cluster 1. These results are in agreement with the general overview of the global functional form. In addition, no plateau was visible. For cluster 3, the picture was quite different, and an L-shape was visible. The initially high values of the OSI for participants with very low PASI declined over PA levels until a value of around 0.5. From this point on, it stays stable or even increases slightly. Finally, for cluster 4, there was an inverted U-shape clearly visible. The initially low OSI increased slightly with the progression of PA to the value of approximately 0.35, after which OSI results steeply declined.

In the last step of the analysis, an attempt to explain cluster membership was attempted. The question was why certain participants displayed some distinct functional form patterns between OSI and PA. The three major functional forms were distinguished: (1) linear progression (which means a decline in balance control with increasing PA), (2) decline with a stable phase, and (3) inverted U-shape.

One-way ANOVA tests were used to assess the role of the calendar and biological age (APHV) and BMI. For qualitative variables such as sex and myopia, χ^2^ tests were used. Results are presented in Table 3.

The mean values of the calendar age in each cluster were: cluster 1: 9.24 (SE: 0.13), cluster 2: 9.36 (SE: 0.12), cluster 3: 9.43 (SE: 0.13), and cluster 4: 9.51 (SE: 0.13). There were no statistically significant differences in calendar age between participants agglomerated into the four clusters (F = 0.76, *p* = 0.516). Thus, calendar age did not play a role in relationship patterns between OSI and PA for the clusters.

The mean values of the APHV in each cluster were: cluster 1: 12.05 (SE: 0.12), cluster 2: 11.91 (SE: 0.11), cluster 3: 12.21 (SE: 0.12), and cluster 4: 12.05 (SE: 0.12). The biological age did not affect the membership in the clusters. Any differences in APHV were not statistically significant (F = 1.22, *p* = 0.308).

The mean values of BMI in each cluster were: cluster 1: 15.50 (SE: 0.52), cluster 2: 16.93 (SE: 0.47), cluster 3: 17.40 (SE: 0.0.51), and cluster 4: 18.95 (SE: 0.53). BMI was the variable that significantly differentiated participants into clusters (F = 7.47, *p* < 0.001). A detailed comparison with Tukey’s HSD post hoc tests revealed significant differences between cluster 1 and cluster 3 (*p* = 0.048), cluster 1 and cluster 4 (*p* < 0.001), and cluster 2 and cluster 4 (*p* = 0.026), whereas there were no significant differences between cluster 1 and cluster 2, cluster 2 and cluster 3, and cluster 3 and cluster 4.

There were 11 boys and 14 girls in cluster 1, and in cluster 2, there were ten boys and 20 girls. In cluster 3, there were 15 boys and 11 girls, and in cluster 4, there were 11 boys and 13 girls. The proportions within the clusters were not statistically significant (χ^2^ = 3.39, *p* = 0.335). Thus, the sex factor did not link patterns of functional forms of the relationship between OSI and PA in clusters.

Finally, myopia was assessed. There were five myopic participants and 20 non-myopic participants in cluster 1, and 7 myopic and 23 non-myopic participants in cluster 2. In cluster 3, there were 18 myopic and 8 non-myopic participants, and in cluster 4, there were 22 myopic and 2 non-myopic participants. The proportions across the clusters were statistically significant (χ^2^ = 42.07, *p* < 0.001). Thus, the myopia factor was very strongly linked with patterns of the functional forms of the relationship between OSI and PA in clusters.

## 4. Discussion

The present contribution demonstrated that functional forms between OSI and PASI differ between children aged 8–11, and four clusters can be identified. The clusters corresponded to the following forms: (1) linear decline, (2) L-shape—linear decline with steady phase, and (3) inverted U-shape—increases at the beginning, a peak value, and a decline from the peak value together with increases in PASI. The testing differences for explanatory variables—sex, myopia, calendar age, biological age, and BMI—revealed that some significant effects were present. Myopia and BMI turned out to be useful in explaining cluster memberships, whereas the rest (sex, calendar age, and biological age) were not. Children in all clusters were on average the same calendar age. An insignificant trend emerged in biological age, with the highest average maturation of children in cluster 3 and the lowest average maturation in cluster 2.

Similarly, an insignificant trend in sex was observed, with fewer boys than girls in clusters 1 and 2 and more boys than girls in clusters 3 and 4. Meanwhile, the distribution of myopic children in the clusters was significant. Most of the myopic children were in cluster 3 and especially in cluster 4, whereaws non-myopic children were members of clusters 1 and 2. Children from clusters 3 and 4 had significantly higher BMI values than children from clusters 1 and 2.

A linear constant–decline functional form of the relationship between balance control and PA volume suggested a small improvement in balance control as PA increases. This relationship seems to dominate in non-myopic, thin, and later-maturated girls. L-shape functional forms suggest an optimal value for PA level for balance-control improvement. Reaching this specific threshold gives no further improvement in balance control. This kind of functional form in the relationship between balance control and PA was distinct for myopic, early-maturated boys with a BMI slightly over average values. Finally, the inverted U-shape form of the relationship between balance and PA suggests the small importance of PA on balance control to a certain level of PA volume (peak value). However, increasing the PA beyond this specific threshold improves balance control. This kind of relationship pattern was found mainly for myopic boys and girls with rather high BMIs compared to peers and who maturated normally.

Our results suggest that myopia is significantly linked with functional forms in the relationship between OSI and PA in the clusters, which means that myopic children demonstrate different functional forms in the relationship between PA and balance control than non-myopic children. Some previous studies have shown an inverse association between myopia and PA in school children compared to their non-myopic peers [22,23]. For instance, in a prospective study among 12-year-old children, Deere et al. [22] observed that compared to non-myopic peers, myopic children were less active (*p* = ≤ 0.001), spent less time in moderate to vigorous PA (*p* = 0.003), and had more sedentary time per day (*p* = 0.002). Lower engagement in PA may have negative consequences on the functional status of children with myopia [26], and myopia could negatively affect the children’s functional status in balance tasks [52,53]. During healthy development, as children mature, balance control increases [54]. In young children, postural sway is more influenced by visual manipulation and has a poorer sensory adaptation than older children and adults [55]. Additionally, the developmental level is considered to be a much better predictor of balance-control improvement than CA [56]. However, a considerable disagreement still exists on whether the relationship between balance control and PA occurs linearly or non-linearly, and whether a sensory period in this relationship can be identified [57]. The results of our study confirm the complexity of this issue, as neither calendar nor biological age explained cluster membership. On the contrary, Kolic et al. [58] observed an improvement in balance with CA from four to twelve. In addition, girls demonstrated more mature balance strategies at earlier ages, which could suggest a relationship between biological age and balance control [3,59]. However, the intensity of the changes decreased in the older groups. Perhaps this could explain our results, taking into account the narrow range of CA. Further, other results have shown that developmental level appeared to be a much better predictor of balance improvement than CA [56]. What is more, authors have observed significant changes between children with different biological ages until the prepuberty period (the most intense in preschool children). Therefore, our consistent results suggest a lack of chronological and biological age impact on balance control. Correspondingly, other older studies reported that balance performance matured at around ten years of age, which is in line with our findings [60,61].

Moreover, PA is considered an independent environmental factor concerning the development and progression of myopia [62]. Outdoor PA especially leads to a lower incidence of myopia and its progression in children, by 13% to 50% [21,63]. Thus, unfortunately, myopic children with limited PA may have a decline in motor skill competency and, at the same time, be more at risk of having increased visual impairment. Our study results showed that improvements in balance control in myopic children require a specific dose of PA for myopic children (Figure 3), and importantly, this health-related factor (myopia) and fundamental motor skill (lower balance control) may cause a further decline in their PA engagement [6,9].

The second significant factor related to the different functional forms in the relationship between balance control and PA was BMI as a relative body weight measurement. Previous studies have shown a higher BMI value among children with lower levels of PA [64,65], with a rather non-linear pattern. Recently, Jago et al. [66] observed that PA decreased and sedentary behavior increased on average for all children between ages 6 and 11. After age six, the differences in PA levels deepened gradually between children in the higher BMI category (overweight/obese) and children with normal values of BMI. Moreover, it has been indicated that a lower PA in 7–15-year-olds was associated with increased BMI, and the most sensitive period with the largest increase in adiposity associated with sedentary behavior was noted over the 9–12-year-old period [64].

The differences in PA between BMI categories increase over time, becoming more highlighted as children age and their functional status develops. Experimental studies have shown that obese children had more postural sway during balance testing across different stability levels compared to children of normal weight [67]. The functional limitations imposed by a higher BMI are caused by decreased mechanoreceptor functions in the feet, reduced relative muscular strength, and decreased muscular fatigue resistance, leading to motor delays and insufficient corrective torque [68]. The current study results showing an inverted U-shape for the relationship between balance and PA were peculiar, mainly for myopic children with high BMI. These findings are important for creating potentially helpful programs to change sedentary behaviors for myopic schoolchildren. The identified types of relationships are useful for developing activity programs that consider a child’s needs resulting from their biological, motor, and health determinants and to develop strategies to prevent decreases in their functional status.

## 5. Limitations

Some limitations are present in our study. First, the statistical approach is quite different than that usually conducted, and several competing techniques exist. This may cause difficulty with comparisons and discussing results. Second, all analyses were based on cross-sectional data using a parental report questionnaire, leading to inflated activity estimates. This relates to the next limitation, which was the necessity of using a general synthetic index (PASI), which combined several determinants of PA into one common index. Using MET values calculated from the International Physical Activity Questionnaire (IPAQ) would indicate real, specific thresholds (volume of PA) for determination, such as plateau or peak values in L-shape or U-shape functional forms describing the relationship between balance control (or other DV) and PA. Although power analysis suggested 105 participants were sufficient for the analysis, it was supposed to determine equal numbers in the groups. However, clusters were not equal to the number of individuals contained in each cluster. This could have an impact on the results of the comparisons. In further research, using the k-mean cluster analysis variant may be more justified.

Our results can be generalized to children in the same range of age and living in urban regions. However, further research is needed to determine the extent to which our results can be generalized (1) to children living in other settings, (2) to specific subgroups of young patients with other visual impairments that reduced balance control, such as strabismus and amblyopia [69,70], and (3) to other measures of PA.

## 6. Conclusions

This contribution, which attempts to identify the effects of PA on balance control, found that the functional form of the relationship is more curvilinear than linear, and the model with the second term of PA explained the best relationship between balance control and PA. However, detailed analysis showed that the functional form of the relationship is related to a child’s BMI and myopia presence and, to a lesser extent, to sex and biological maturation. Three functional forms of the relationship were identified: linear constant-decline (which means constantly improving balance control together with increased PA volume), distinctive for non-myopic girls with low BMI and insignificantly less maturated; L-shape (which means that there is a plateau in PA for balance control development) distinctive for myopic, early-maturated, and boys with BMI slightly above average; inverted U-shape (which means a peak value of the PA that is the threshold for the volume of PA when stimulating effect starts) distinctive for myopic boys and girls with rather high BMIs compared to their peers, but maturing at an average pace. Future studies should be focused on evaluating the causal relationship between balance control and PA using other explanatory variables, e.g., socioeconomic status.

## Figures and Tables

**Table 1:** Demographic and clinical characteristics of the children.

Parameters	Myopic Children		Non-Myopic Children	
	Mean	SD	Mean	SD
Age (years)	10.14	1.24	10.54	1.36
Age at peak height velocity (years)	12.17	0.60	11.93	0.57
Height (cm)	139.94	6.39	139.30	5.48
Body mass (kg)	36.15	8.06	31.21	4.57
Body Mass Index (kg/m^2^)	18.33	3.28	16.02	1.59
Overall Stability Index (°)	2.11	1.04	0.98	0.54
Physical Activity Synthetic Index (pts)	0.421	0.07	0.475	0.09
Distance visual acuity (RE)	0.86	0.24	1.00	0.04
Distance visual acuity (LE)	0.91	0.21	1.00	0.03
Visual acuity (near)	0.50	0.01	0.50	0.01
Spectacle correction (RE) (D)	0.97	0.10	1.00	0.00
Spectacle correction (LE) (D)	1.00	0.03	1.00	0.00
Spectacle correction (mean of both eyes) (D)	0.98	0.51	1.00	0.00
Spherical equivalent (RE) (D)	−2.08	1.38	0.17	0.11
Spherical equivalent (LE) (D)	−2.14	1.74	0.23	0.05
Spherical equivalent (mean of both eyes) (D)	−2.16	1.51	0.05	0.27
Spherical equivalent before the cycloplegia (RE) (D)	−1.89	1.37	0.16	0.22
Spherical equivalent before the cycloplegia (LE) (D)	−1.97	1.71	0.12	0.13
Spherical equivalent before the cycloplegia (mean of both eyes) (D)	−1.93	1.44	0.14	0.17

Note: pts—points, RE—right eye, LE—left eye, D—dioptres.

**Table 2:** Regression results for the entire sample (boys and girls) (*p*-value for R^2^).

Statistic	Function		
	F1	F2	F3
PA	−3.40 *	−7.27	21.15
PA^2^		4.13 *	−57.63
PA^3^			43.02
Constant	3.07 *	3.94 *	−0.247
R^2^	0.080 *	0.092 *	0.069 *

Note: * *p* < 0.05.

**Table 3:** Comparison of the clusters regarding age, age at peak height velocity, body mass index, sex, and presence of myopia.

Variable		1	2	3	4	*p*
		Mean (95% CI)	Mean (95% CI)	Mean (95% CI)	Mean (95% CI)	
CA		9.24 (8.99–9.49)	9.36 (9.13–9.59)	9.43 (9.18–9.68)	9.51 (9.25–9.76)	0.516
APHV		12.05 (11.82–12.29)	11.91 (11.69–12.12)	12.21 (11.98–12.44)	12.05 (11.80–12.29)	0.308
BMI		15.50 (14.47–16.52)	16.93 (15.99–17.86)	17.40 (16.39–18.40)	18.95 (17.90–19.99)	<0.001 *
sex	boys	11	10	15	11	0.335
	girls	14	20	11	13	
myopia	yes	5	7	18	22	<0.001 *
	no	20	23	8	2	

Note: CA—calendar age, APHV—the age at peak height velocity, BMI—body mass index. * *p* < 0.05.

## Data Availability

The data presented in this study are available upon request from the corresponding author.
